# A new southern distribution limit for *Sorex mirabilis* Ognev, 1937 (Eulipotyphla, Soricidae)

**DOI:** 10.1080/19768354.2018.1561515

**Published:** 2019-01-03

**Authors:** Sung Jin Park, Woo-Shin Lee

**Affiliations:** a Department of Forest Sciences, CALS, Seoul National University, Seoul, Korea; b Research Institute of Agriculture and Life Sciences, CALS, Seoul National University, Seoul, Korea

**Keywords:** eoreumgol, ice valley, Ussuri shrew

## Abstract

*Sorex mirabilis*, a small insectivorous mammal, is a very rare, poorly known species distributed at high latitudes and altitudes of northeastern Asia. The occurrence of this species in the Republic of Korea was known from late 1990s. We found a new specimen from a location of a 50′ lower latitude and 400 m lower altitude than the previously known locations of the first record in the Republic of Korea. This finding may imply that *S. mirabilis* is distributed in a wider range than previously considered and that their distribution might be related to eoreumgols, or ice valleys, where there are potential insular refugia due to micro-meteorological characteristics.

## Introduction

The Ussuri shrew, also known locally as the giant shrew (*Sorex mirabilis* Ognev 1937), is a Palaearctic species with a distribution restricted to northeastern Asia (Ellerman and Morrison-Scott 1966; Won 1968; Corbet 1978; Nowak 1999; Yoon et al. 2004; Wilson and Reeder 2005). It was known that *S. mirabilis* was distributed in northeastern China, the Ussuri region (Russia), and North Korea (Won 1968; Stone 1995; Nowak 1999), but in late 1990s the occurrence of this species was reported in South Korea from one specimen from Mt. Jeombong (or Chombong, 800 m alt., 38°02′35″N, 128°25′40″E) in 1997 and two specimens from Mt. Odae (850 m alt., 37°47′03″N, 128°34′23″E) in 1999 (Han et al. 2000, Figure 1). This shrew species is very rare and poorly known, and it is categorized as Data Deficient in the IUCN Red List (Tsytsulina 2008); therefore, it is important to accumulate more distribution information. Here, we report a new southern and lower elevational record of *Sorex mirabilis* and discuss its potential habitat associations in Korea.

**Figure 1.**
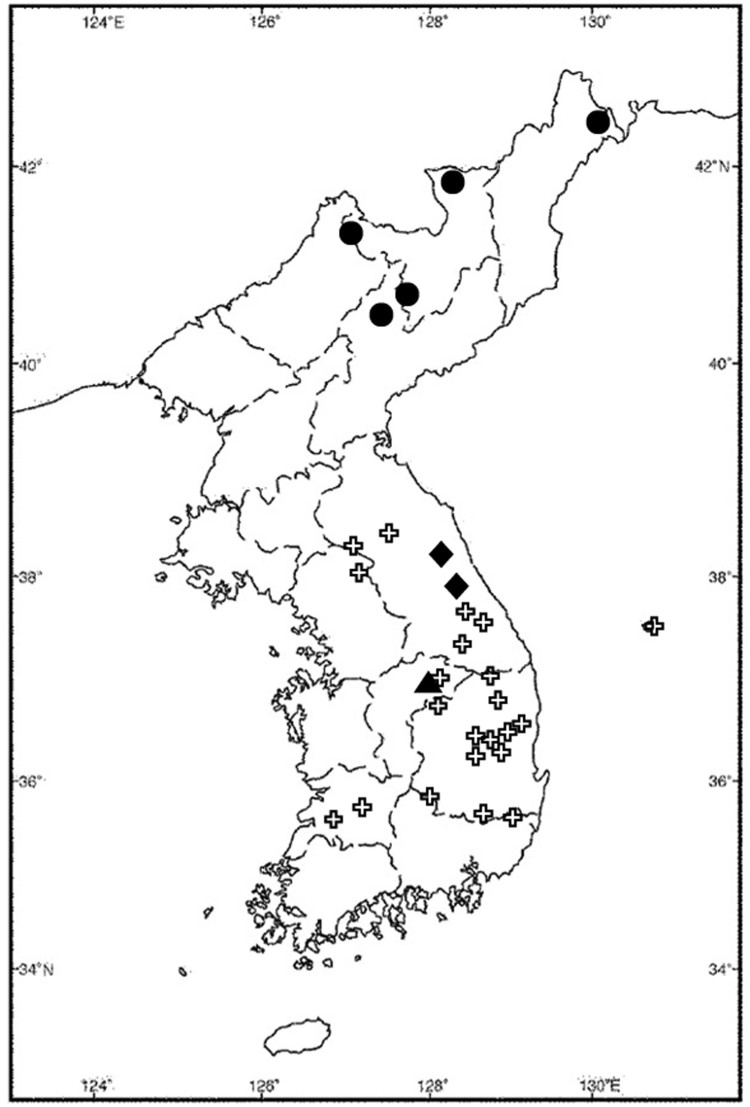
Distribution map of the records of *Sorex mirabilis* in the Korean Peninsula. Circles denote records in North Korea (MAB National Committee of DPRK 2002); diamonds denote the first records in South Korea (Han et al. 2000); triangle denotes the new southern-most record (authors’ data); crosses denote eoreumgols (ice valleys) (Kim 2005).

## Materials and methods

**Figure 2.**
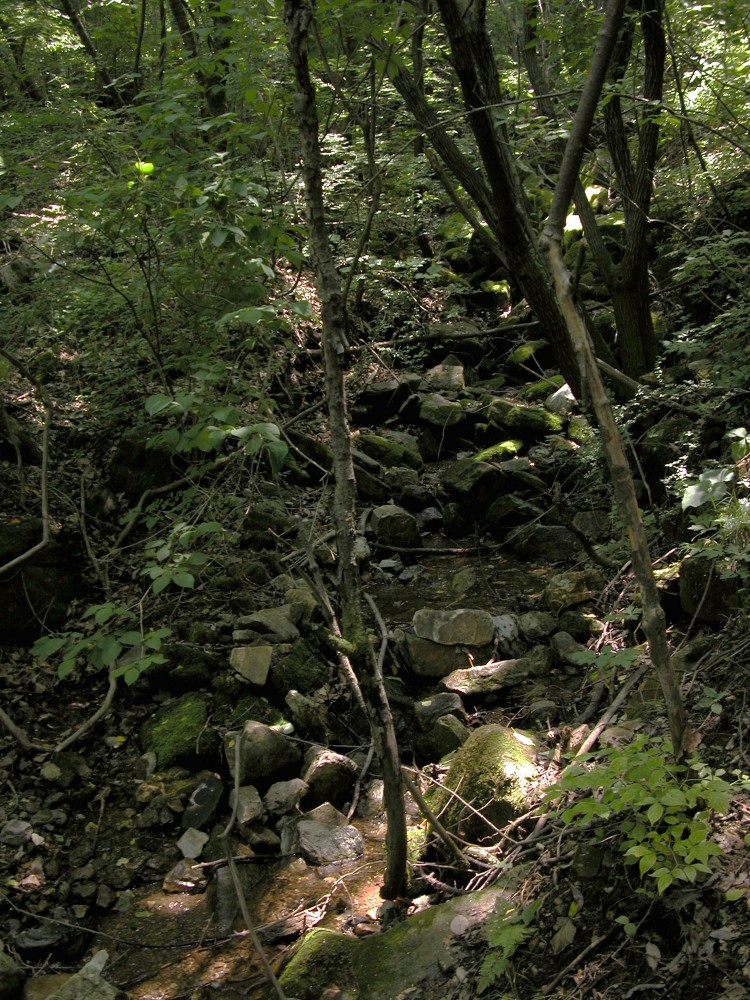
Capture site of *Sorex mirabilis* in Mt. Malmok, Jeoksung-myeon, Danyang-gun, Chungcheongbukdo, the Republic of Korea (36°57′14.6″N, 128°16′34.6″E, 400 m elevation, northern aspect).

We set up 200 Sherman large folding aluminum live traps (23 × 8.9 × 7.6 cm) with roasted peanut baits and checked the traps for 10 nights from 9 July to 18 July 2006 at Mt. Malmok, Jeoksung-myeon, Danyang-gun, and Chungcheongbuk-do in the Republic of Korea (Figure 1, 36°57′14.6″N, 128°16′34.6″E, 400 m elevation, northern aspect). The capture site was a rocky hillside in a coniferous-broadleaf mixed forest (Figure 2). The overstory was dominated by *Pinus densiflora*, *Quercus mongolica,* and *Purus sergeant*. *Q. mongolica, Q. serrata,* and *P. densiflora* were the dominant species of the midstory. The lower story was dominated by *Rhododendron mucronulatum, Rhus trichocarpa*, *Fraxinus sieboldiana,* and the herbaceous plants were *Disporum smilacinum, Ainsliaea acerifolia*, *Astilbe chinensis, Carex humilis, Smilax sieboldii,* and *Carex okamotoi*. There was no valley or stream, but water seeped from a gap of rock cover onto the forest floor on the capture date during the rainy season (Figure 2). The annual mean temperature in this area is 10.1°C, with a maximum temperature of 29.2°C in August, a minimum temperature of −11.1°C in January, and an annual mean precipitation of 1,295 mm concentrated from late June to July (data from the Jecheon observation station of the Korea Meteorological Administration, 24 km north of the trapping site).

On 15 July 2006, we caught one dead *S. mirabilis* at the trapping site. Body measurements (total length, tail length, and foot lengths) of our specimen were measured with a ruler and body weight with an electronic scale (Casbee, MW-1200, 0.1 g unit). A dial caliper (Mitutoyo, 505–666, 0.01 mm unit) was used for skull measurements. The skull specimen of *S. mirabilis* was deposited at the Wildlife Laboratory of the Department of Forest Sciences at Seoul National University (Registration No: 2006-S-1). During trapping 23 *Apodemus agrarius,* 12 *A. peninsulae,* 10 *Myodes regulus*, and 2 *Tamias sibiricus* were also captured. No other insectivores were caught probably due to a vegetable type of bait, roasted peanut, we used.

## Results and discussion

### Description

**Figure 3.**
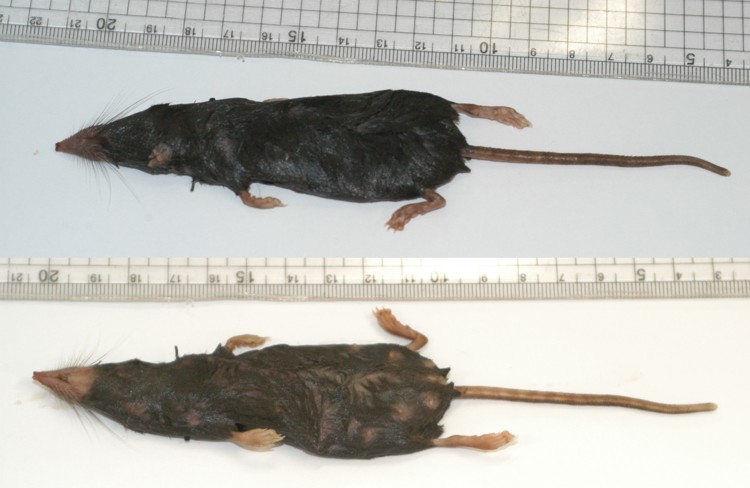
Dorsal and ventral appearance of *Sorex mirabilis*.

The specimen of *S. mirabilis* was an adult female with three inguinal pairs of developed nipples and a robust tail (Figure 3). Inspection of the uterus was impossible to check pregnancy because of putrescent state of the specimen, though she did not have a conspicuously large abdomen.

**Figure 4.**
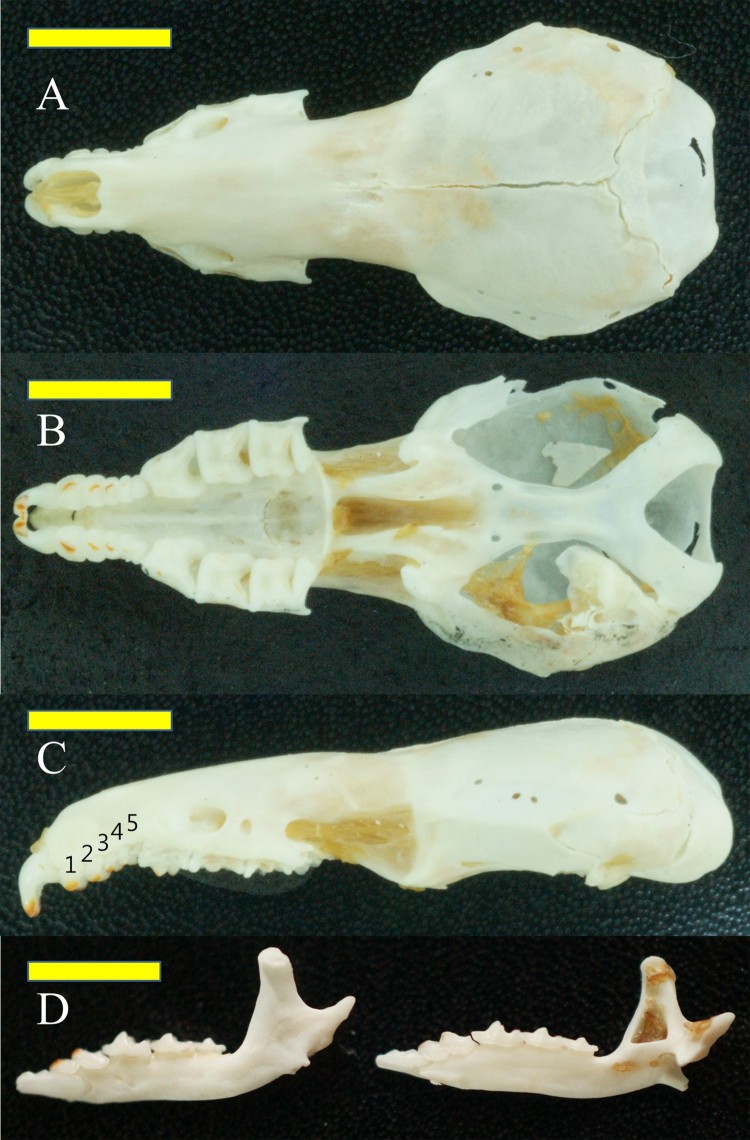
*Sorex mirabilis* skull. A: dorsal view, B: view of the upper teeth and palate. C: lateral left view of the unicuspids on the upper jaw, with numbers indicating the order of unicuspids. D: lateral and medial views of the mandibles. Horizontal bars = 5 mm.

Eleven species of Soricidae have been recognized in the Korean Peninsula: 3 *Crocidura* species, 1 *Neomys* species, and 7 *Sorex* species (NIBR 2011). Identification of *S. mirabilis* was made on the diagnostic features as follows. First, we confirmed the specimen as *Sorex* species with pigmented teeth of a dental formula of I 3/1, C 1/1, P 3/1, and M 3/3, including 5 unicuspid teeth from I^2^ to P^2^ (Ohdachi et al. 2009). Then, size measurements confirmed it as *Sorex mirabilis*. *S. mirabilis* is the largest *Sorex* species in Asia (Han et al. 2000). Their total length is greater than 120 mm, with a large skull size with the greatest length over 22 mm and the hind foot greater than 16 mm. Other 6 *Sorex* species occurring in Korea have shorter total length and skull size (Jones and Johnson 1960; Ellerman and Morrison-Scott 1966; Won 1968; Corbet 1978; Hoffmann 1987; Han et al. 2000; Yoon et al. 2004; Smith and Xie 2008). Dentition reconfirmed the identification. The anterior cusp of the first upper incisor of *Sorex mirabilis* bends deeply and the posterior cusp of the first upper incisor is very small and barely projects (Corbet 1978; Han et al. 2000), while other *Sorex* shrews occurring in Korea have predominant posterior cusps (see figures in Ohdachi and Maekawa 1990; Han et al. 2000; Ohdachi and Han 2005; Cook et al. 2016). The third upper unicuspid of *S. mirablis* is usually much smaller than the fourth unicuspid (Jones and Johnson 1960; Ellerman and Morrison-Scott 1966; Corbet 1978; Han et al. 2000; Smith and Xie 2008). However, the size of the third upper unicuspid of our specimen was similar to the fourth (Figure 4). This might be due to heavier wear on the fourth unicuspid than the third upper unicuspid; red pigment on the fourth upper unicuspid was barely observed compared with the first, second, and third upper unicuspids, indicating that the fourth upper unicuspid was heavily worn. The details of the external and cranial measurements, taken with a dial caliper, are shown in Table 1.

**Table 1.** External, cranial, and dental measurements (in mm and gram) of the specimen of *Sorex mirabilis*.

Sex	BW	TL	T	FF(cu)	HF(cu)	HF(su)	E	GL	WB	MB	UTRL	Remark	Source
Female	15.2	165	68	11.7	17.0	-	8.0	24.02	10.75	6.72	10.25		Authors’ data
Unknown	–	145	68	11.6	17.7	15.2	–	23.50	10.78	–	–		Han et al. (2000)
Female	15.7	171	73	11.7	17.0	15.6	10.5	22.90	10.73	–	–		
Male	15.3	158	69	11.8	17.7	16.2	–	23.45	10.77	–	–		
Male	12.0	160.9	71.8	–	–	16.5	9.0	–	–	–	–		Won (1968)
Female (*n* = 12)	12.6	157.6	75.1	–	16.7	16.7	8.3	23.70	–	–	–	mean value	

BW: body weight, TL: total length, T: tail length, FF (cu): forefoot length with claws, HF (cu): hind foot length with claws, HF(su): hind foot length without claws, E: Ear, GL: greatest length of skull, WB: width of brain case, MB: maxillary breadth, UTRL: upper tooth row length.

### Distribution

Won (1968) noted that *S. mirabilis* is likely distributed only at high elevations on the Korean Peninsula. The specimens of Han et al. (2000) were also from high elevations (800–850 m). However, our finding extends the known distribution to a latitude that is 50′ farther south and at an elevation that is 400 m lower than previously known. This may imply that *S. mirabilis* is distributed in a wider range than previously considered, although this species may be very rare.

Unique microhabitat of the capture site also may relate to the occurrence of *S. mirabilis*. Our capture site is 6 km away from the eoreumgol of Mt. Geumsu; eoreumgols may be a possible reason for a farther south and lower-elevation distribution compared with the previously known distribution of *S. mirabilis*. Eoreumgols, or ice (eoreum) valleys (gol), are so named because cold winds blow and ice often forms beneath talus slopes in the valleys in summer (Kim 1968). By contrast with the cold microclimate in summer, water does not freeze, or ice melts, in winter in eoreumgols (Byun et al. 2011). This phenomenon has been explained by the effect of water evaporation, underground ice bodies, cold air from the polar region, radiative cooling, and diurnal and seasonal respirations in the talus (Byun et al. 2011).

Due to micro-meteorological characteristics, eoreumgols are regarded as insular refugia for northern or subarctic plant species in Korea (Kim et al. 2006; Kong et al. 2011). Therefore, eoreumgols may also be important refugia for small mammals dwelling in northern latitudes and high altitudes of Korea, such as *S. mirabilis* in this case. Approximately 20 eoreumgols are known in South Korea (Kim 2005), and many of them are located in areas farther south than the known locations of *S. mirabilis* specimens, including ours (Figure 1). Further exploration of eoreumgols is required to expand our knowledge of the distributions of subarctic small mammals in Korea.

## References

[CIT0001] Byun HR, Tanaka HL, Choi PY, Kim DW. 2011 Seasonal reversal at Miryang Eoreumgol (Ice valley), Korea: observation and monitoring. Theor Appl Climatol. 106:403–415. doi: 10.1007/s00704-011-0429-5

[CIT0002] Cook JA, McLean BS, Jackson DJ, Colella JP, Greiman SE, Tkach VV, Jung TS, Dunnum JL. 2016 First record of the Holarctic least shrew (*Sorex minutissimus*) and associated helminths from Canada: new light on northern Pleistocene refugia. Can J Zool. 94:367–372. doi: 10.1139/cjz-2015-0212

[CIT0003] Corbet GB. 1978 The mammals of the Palaearctic region: a taxonomic review. London: British Museum (Natural History).

[CIT0004] Ellerman JR, Morrison-Scott TCS. 1966 Checklist of Palaearctic and Indian mammals, 1758 to 1946, 2nd ed London: British Museum (Natural History).

[CIT0005] Han SH, Ohdachi S, Abe H. 2000 New records of two *Sorex* species (Soricidae) from South Korea. Mamm Study. 25:141–144. doi: 10.3106/mammalstudy.25.141

[CIT0006] Hoffmann RS. 1987 A review of the systematic and distribution of Chinese red-toothed shrews (Mammalia: Soricinae). Acta Theriol Sinica. 7:100–139.

[CIT0007] Jones JK Jr., Johnson DH. 1960 Review of the insectivores of Korea. Univ Kans Publ, Mus Nat Hist. 9:549–578.

[CIT0008] Kim SS. 1968 On the ice-formation at the “Ice-valley” Milyang Koon, Korea in summer season. J Korean Meteorol Soc. 4:13–18. Korean.

[CIT0009] Kim YY. 2005 On the geographical features and ice-formation at Korean Ice-valley. Asia-Pac J Atmos Sci. 41:1151–1161. Korean.

[CIT0010] Kim JS, Chung JM, Lee BC, Pak JH. 2006 The plant species composition and phytogeographical significance of algific talus slope in Korea. Kor J Plant Taxon. 36:61–89. Korean. doi: 10.11110/kjpt.2006.36.1.061

[CIT0011] Kong WS, Lee S, Yoon K, Park H. 2011 Environmental characteristics of wind-hole and phytogeographical values. J Environ Impact Assess. 20:381–395. Korean.

[CIT0012] MAB National Committee of DPRK 2002 Red data book of DPRK (Animal). Pyongyang, DPR Korea: MAB National Committee Academy of Sciences.

[CIT0013] NIBR 2011 National list of species of Korea (vertebrates). Incheon, Korea: National Institute of Biological Resources Korean.

[CIT0014] Nowak RM. 1999 Walker’s mammals of the world Volume II, 6th ed Baltimore and London: The Johns Hopkins University Press.

[CIT0015] Ognev SJ. 1937 A new and remarkable species of shrew (*Sorex mirabilis sp. nova*). Bull Soc Nat Moscou S Biol. 46:268–271. Russian.

[CIT0016] Ohdachi SD, Han SH. 2005 Records of *Sorex* species (Soricidae, Mammalia) from Mt. Paektu, North Korea, with the first record of S. daphaenodon. Eurasian J. For. Res. 8–2:71–73.

[CIT0017] Ohdachi SD, Ishibashi Y, Iwasa MA, Saitoh T, editors. 2009 The wild mammals of Japan. Kyoto: Shoukadoh.

[CIT0018] Ohdachi S, Maekawa K. 1990 Relative age, body weight, and reproductive condition in three species of *Sorex* (Soricidae; Mammalia) in Hokkaido. Res Bull Coll Exp For Hokkaido Univ. 47:535–546.

[CIT0019] Smith AT, Xie Y, editors. 2008 A guide to the mammals of China. Princeton and Oxford: Princeton University Press.

[CIT0020] Stone RD. 1995 Eurasian insectivores and tree shrews: status survey and conservation action plan. Gland: IUCN.

[CIT0021] Tsytsulina K. 2008 *Sorex mirabilis*. IUCN Red List of Threatened Species Version 2014.2. http://www.iucnredlist.org. Downloaded on 22 October 2014.

[CIT0022] Wilson DE, Reeder DM. 2005 Mammal species of the world: a taxonomic and geographic reference, 3rd ed Baltimore, MD: Johns Hopkins University Press.

[CIT0023] Won HG. 1968 Mammals in Chosun. Pyongyang, North Korea: Kwahakwon Press Korean.

[CIT0024] Yoon MH, Han SH, Oh HS, Kim ZG. 2004 The mammals of Korea. Seoul: Dong-bang Media Korean.

